# Active myeloperoxidase: a promising biomarker to differentiate “acute” and “low-grade” peri-prosthetic joint infections from aseptic failures

**DOI:** 10.3389/fmicb.2024.1417049

**Published:** 2024-06-07

**Authors:** Martina Maritati, Giuseppe De Rito, Valentina Rosta, Carlo Cervellati, Maria Cristina Manfrinato, Gustavo Alberto Zanoli, Roberto De Giorgio, Matteo Guarino, Anna Costanzini, Carlo Contini, Yu Ning, Andrej Trampuz, Alessandro Trentini

**Affiliations:** ^1^Center for Musculoskeletal Surgery, Charité—Universitätsmedizin Berlin, Corporate Member of Freie Universität Berlin, Humboldt-Universität zu Berlin, and Berlin Institute of Health, Berlin, Germany; ^2^Department of Translational Medicine, University of Ferrara, Ferrara, Italy; ^3^Orthopaedic Ward, Casa di Cura Santa Maria Maddalena, Occhiobello (Rovigo), Italy; ^4^Department of Environmental and Prevention Sciences, University of Ferrara, Ferrara, Italy; ^5^Department of Neuroscience and Rehabilitation, University of Ferrara, Ferrara, Italy; ^6^Department of Medical Sciences, University of Ferrara, Ferrara, Italy

**Keywords:** periprosthetic joint infection, active myeloperoxidase, synovial biomarker, neutrophil extracellular trap, aseptic failure, low grade periprosthetic joint infection

## Abstract

**Introduction:**

The accurate distinction between periprosthetic joint infections (PJI) and aseptic failures (AF) is of paramount importance due to differences in treatment. However, this could be challenging by using the current criteria. Various synovial fluid biomarkers are being assessed to improve the diagnostic accuracy. Myeloperoxidase (MPO), an enzyme contained in the granules of neutrophils, may be a promising biomarker for PJI.

**Methods:**

Synovial fluids of 99 patients (*n* = 65 PJI according to EBJIS criteria; *n* = 34 AF) were collected in two specialized orthopedic centers. PJI were divided into acute (*n* = 33) and low-grade (*n* = 32) according to previously published classification. An activity assay specific for active MPO was performed in each sample. Ability of MPO to correctly discriminate patients with PJI from AF was determined by ROC analysis. The best discriminating cut-off value was determined by calculating the J Youden index. For all analyses, a *P* value < 0.05 was considered statistically significant.

**Results:**

Active MPO was higher in PJI than AF (*P* < 0.0001). The ROC analysis revealed a significant area under the curve (AUC: 0.86; 95% CI: 0.78–0.93, *P* < 0.0001). A cut-off value of 561.9 U/mL, with good sensitivity (0.69) and specificity (0.88), discriminated between AF and PJI (accuracy 75.76%, 95% CI: 66.11–83.81%, positive likelihood ratio 5.88, 95% CI: 2.31–14.98 and negative likelihood ratio 0.35, 95%CI: 0.24–0.51). No difference in MPO levels was found between acute and chronic low-grade PJI.

**Conclusion:**

The proposed assay appears to be a reliable and affordable tool for detecting the active MPO in synovial fluid, with promising characteristics of sensitivity and specificity in discriminating both acute and low-grade PJI from AF. Further studies are needed to confirm MPO diagnostic cut-off values and validate their use in the routine clinical practice.

## 1 Introduction

Periprosthetic joint infections (PJI) represent one of the most dreaded complications in arthroplasty, since they are burdened with significant disability for patients and prohibitive costs for national health systems (Patel, 2023). Although considerable efforts have been made to improve the diagnostic accuracy of currently available diagnostic algorithms, a universally recognized gold standard still does not exist (Sigmund et al., 2022).

One of the most reliable and often performed diagnostic procedures is synovial fluid examination with total and differential leucocyte count (Dinneen et al., 2013). However, its specificity is poorer when inflammation is sustained by causes other than infection (Tande and Patel, 2014).

Since the treatment of PJI radically differs from that of aseptic failures (AF), it is crucial to correctly diagnose these two pathological frameworks.

However, a clear classification may not be achieved, especially in “doubtful” cases, which often involve chronic infections characterized by low-grade inflammation (Corvec et al., 2012).

To help decision making in this group, several biomarkers are currently being assessed as alternatives or aids to synovial fluid leucocyte measurement (Vrancianu et al., 2023).

Among these, only alpha defensin was sufficiently investigated to be included in the diagnostic algorithms of the International Consensus Meeting of Philadelphia (ICM) (Parvizi et al., 2018) and, more recently, in the European Bone and Joint Infection Society (EBJIS) criteria (McNally et al., 2021).

**FIGURE 1:**
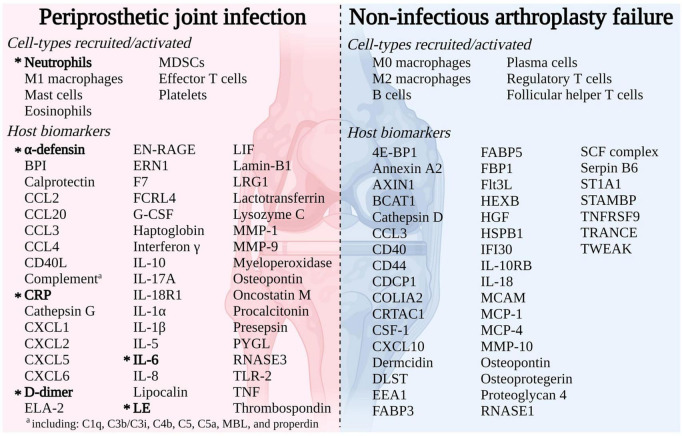
Immune response to arthroplasty failure due to periprosthetic joint infection (PJI) or aseptic failure (AF) detailing cell-types recruited/activated and host markers (Fisher and Patel, 2023).

A thorough description of the host immune response in the PJI and AF microenvironment was outlined thanks to recent developments in “multi-omics” approaches (Figure 1). Myeloperoxidase (MPO), one of the several biomarkers being studied, has recently garnered attention since it is thought to play a role in the development of PJI but not AF (Fisher and Patel, 2023).

Monocytes and neutrophils produce MPO, which is subsequently packaged into azurophilic (primary) granules and either released into the extracellular space or phagosomes (Strzepa et al., 2017). In addition, MPO is a crucial component of neutrophil extracellular traps (NETs), a web-shaped structure supposed to have the ability to kill bacteria and limit their spread (Papayannopoulos, 2018).

In the presence of hydrogen peroxide (H_2_O_2_), the released MPO become active and catalyzes the oxidation of halides and pseudo-halides to form highly oxidizing hypohalous and (pseudo) hypohalous acids. This process increases the toxicity of the reactive oxygen species produced during the respiratory burst against bacteria (Trentini et al., 2020).

Given these premises, the aim of our study was to evaluate the diagnostic potential of active MPO as a marker of PJI. In addition, we sought to verify whether active MPO was differentially expressed between (i) “acute” and “low grade” PJI; (ii) gram-positive- and gram-negative-PJI.

## 2 Materials and methods

### 2.1 Study design

This study represents a two-centers cohort investigation. Frozen synovial fluids of 99 patients together with their demographic and clinical data were collected retrospectively at Charité Center for Musculoskeletal Surgery (Berlin, Germany) and, prospectively, at Casa di Cura Santa Maria Maddalena (Occhiobello, Italy). The data collection period ran from May 2017 to September 2024.

Synovial fluids collected during revision arthroplasties (hip and knee) after ensuring all routine diagnostic examinations were centrifuged for 10 min at 10,000 g and 4°C. Each supernatant was then stored at −80°C until analysis. All patients included in the study provided written informed consent prior to the procedure. Only one sample was collected from patients who underwent several revision surgeries over the study period.

All patients’ demographic information was taken from the electronic medical record, including age, sex, BMI, procedure type (total hip or total knee revision arthroplasty), synovial white blood cell count (cells/μL), and synovial polymorphonuclear cell percentage.

Since MPO is a key enzyme involved in many autoimmune diseases such as psoriatic arthritis, systemic lupus erythematosus, ANCA-associated vasculitis (Li et al., 2022) and rheumatoid arthritis (Stamp et al., 2012), chronic inflammatory joint diseases were an exclusion criterion.

Further exclusion criteria were lack of clinical documentation for retrospectively collected samples, intake of antibiotics and biological drugs at the time of sample collection, insufficient volume of synovial fluid, presence of peri-prosthetic fracture.

This study received approval from the institutional review boards of Charité Center for Musculoskeletal Surgery, Berlin, Germany (Approval Number EA1/026/20) and Casa di Cura Santa Maria Maddalena, Occhiobello, Italy (Approval Number 37370).

The study was conducted in accordance with the Declaration of Helsinki. No funding from external sources was employed to develop this study.

The enrolled patients were divided into two groups (PJI vs. AF) using EBJIS criteria (McNally et al., 2021). Then, PJI were divided into “acute” and “low-grade” according to previously published classification (Izakovicova et al., 2019).

Finally, the category of PJI was divided into two subgroups based on the causative microbiological agent (gram-positive versus gram-negative) to investigate any difference in MPO levels.

The treating physician was not informed about the MPO values, and this did not impact the management of infection.

Finally, the biomarker leucocyte esterase was tested in parallel for comparison.

### 2.2 MPO assay

The assay of MPO activity was essentially carried out as outlined in our previous work (Trentini et al., 2020). Briefly, the wells of an ELISA microplate (Nunc-Maxisorp C-shaped wells, Thermo Scientific, Cat. No. 446612) were coated with 100 μl of anti-MPO polyclonal antibodies (Calbiochem, Cat. No. 475915) diluted 1:500 in 0.2M sodium bicarbonate buffer, pH 9.4, and incubated overnight at 4°C. At the end of the incubation, the wells were washed three times with 300 μl/well of wash buffer (WB, 0.15 M NaCl, 0.1 M NaH2PO4, pH 7.2, 0.05% Tween-20) and the protein-free sites were saturated by incubating the wells with 300 μl of 5% BSA in WB for 1 hour at room temperature with gentle agitation. The plate was subjected to three washing steps with 300 μl/well of WB, and then 100 μl of synovial fluids (diluted 1:4 for AF and 1:300 for PJI samples) or standard (in the range 0.39–25 ng/mL of MPO purified from leukocytes, Calbiochem, Cat. No. 475911) diluted in 1% BSA in WB without Tween-20 (dilution buffer) were dispensed in duplicate in the wells. After 1 hour of incubation at room temperature with gentle agitation and four washing steps with 300 μl/well of WB, each well was dispensed with 50 μl of 392 μM H2O2 (the final concentration in the assay was 196 μM) and 50 μL of 200 μM AmpliFlu Red (Sigma-Aldrich, Cat. No. 90101, prepared from a 200 mM stock solution in DMSO and stored in aliquots at -20 °C; the final concentration in the assay was 100 μM), both diluted in 20 mM citrate buffer, pH 6, containing 80 mM NaBr. The fluorescence of the product (resorufin) was recorded at the excitation wavelength of 535 and emission wavelength of 590 nm every 30 s for 10 min at 37 °C with a microplate fluorimeter (Tecan Infine M200, Tecan, Switzerland). The relative fluorescence units obtained were converted into U/ml of active enzyme according to a standard curve made by different concentrations of resorufin as previously detailed (Trentini et al., 2020). The performance of the assay with the new matrix (synovial fluid) was in line with previous data and showed an intra-assay variability of 6.0% ± 1.5% and an inter-assay variation of 9.3% ± 3.2%.

### 2.3 Leukocyte esterase (LE) assay

The quick test leukocyte esterase (elastase) assay was performed using commercially available urine strips according to Li et al. (2017). Accudoctor strips (MedNet EC-REP, Germany) were used for the test. Briefly, a drop of synovial fluid was dispensed onto the strip, and the result reading was taken after 2 min of incubation at room temperature. Test positivity was confirmed if a dark purple (+++) color developed. Because of the presence of contaminating red blood cells (RBCs) and because centrifugation was performed only after the first freeze/thaw cycle of the samples, we could perform the analysis only in a small subset of clear samples (*n* = 13 AF and *n* = 18 PJI). The test was performed in duplicate by two different operators blinded to the diagnosis. Discrepancies between the two readings were resolved by a third operator.

### 2.4 Statistical analysis

Normality of distribution was checked by the Shapiro-Wilk test. Since the normality was not assumed for all the analyzed variables, data were presented as median (interquartile range). Given the non-parametric nature of data, group comparisons were performed by Mann-Whitney U test. Categorical variables were reported as frequencies and percentages and were compared by Fisher’s exact test.

The influence of covariates such as age, sex, BMI, site of arthroplasty on MPO levels was determined by ANCOVA (analysis of covariance).

The ability of MPO to discriminate between PJI and aseptic revisions was determined by ROC analysis, where the best discriminant cut-off value was calculated by the Youden’s J index (sensitivity + specificity−1). The cut-off value of MPO activity was then used to calculate the positive and negative likelihoods as well as the accuracy of the discrimination.

A *P* < 0.05 was considered statistically significant. All analyses were performed by SPSS 26 (IBM) for Windows. The figures were prepared with GraphPad Prism v9.

## 3 Results

### 3.1 Patient’s population

Of the 99 patients enrolled, 65 (65.6%) were diagnosed with PJI according to EBJI criteria (Category Infection Confirmed) (McNally et al., 2021). Thirty-four (34.4%) patients were considered not infected (Category Infection Unlikely).

Demographic and clinical characteristics of the whole study court and microbiological isolates obtained in PJI are shown in Tables 1, 2, respectively. Patients were not different in age, BMI and sex prevalence, and the site of arthroplasty (hip/knee) was consistent between the two groups (see Table 1). Regarding microbiological isolates, the 20% were cultures negative-PJI whereas the remaining ones disclosed microbiological isolates. Among these, most aetiologic agents were gram-positive pathogens (Table 2, 73.1%), and the remaining were gram-negative (25%) or fungi (1.9%, determined by *Candida glabrata*). Within the gram-positive, *Staphylococcus aureus* was the most frequent isolated bacteria (47.4%) followed by *Staphylococcus epidermidis* (28.9%) and *Staphylococcus hominis* (15.4%). Whitin the gram-negative, *Enterobacter cloacae* and *Serratia marcescens* were the most frequent bacteria (23.1% each), followed by *Escherichia coli* (15.4%).

**TABLE 1:** Clinical and demographical data of the population included in the study.

Variable	AF (*n* = 34)	PJI (*n* = 65)
Sex (*n* females, %)	18 (52.9)	31 (47.7)
Age (years)	73 (68–77)	74 (66–82)
BMI (Kg/m^2^)	28.4 (25.9–32.2)	29.9 (27.4–38.9)
**Site of arthroplasty, *n* (%)**		
Hip	7 (20.5)	25 (35.5)
Knee	27 (79.5)	40 (64.5)
MPO (U/ml)	6.83 (0.43–220.54)	9967.32 (245.53–14476.54)

*Values are expressed as median (interquartile range) or frequencies and percentages where appropriate. AF, Aseptic failure; PJI, prosthetic joint infections; BMI, body mass index; MPO, myeloperoxidase.

**TABLE 2:** Profile of microorganisms isolated from culture-positive patients with PJI (*n* = 65).

Microorganism group	Total joint (*n* = 65)
Negative isolates	13
Gram-positive	38 (73.1)^a^
*Staphylococcus aureus*	18 (47.4)
*Staphylococcus epidermidis*	11 (28.9)
*Propionibacterium acnes*	1 (2.6)
*Staphylococcus caprae*	1 (2.6)
*Streptococcus agalactiae*	2 (5.3)
*Staphylococcus hominis*	1 (2.6)
*Staphylococcus warneri*	1 (2.6)
*Streptococcus disgalactiae*	2 (5.3)
*Streptococcus mitis*	1 (2.6)
Gram-negative	13 (25.0)^a^
*Enterobacter cloacae*	3 (23.1)
*Escherichia coli*	2 (15.4)
*Klebsiella pneumoniae*	1 (7.7)
*Proteus mirabilis*	1 (7.7)
*Pasteurella multocida*	1 (7.7)
*Serratia marcescens*	3 (23.1)
*Staphylococcus hominis*	2 (15.4)
Fungal organism	1 (1.9)
*Candida glabrata*	1 (100.0)

Data are *n* (%). PJI, Prosthetic joint infection.

^a^Percentage with respect to the positive isolates (*n* = 52). Percentages of the single isolates are calculated to the respective total gram-positive or gram-negative isolates.

### 3.2 Active MPO in subjects with aseptic failure and prosthetic joint infection

**FIGURE 2:**
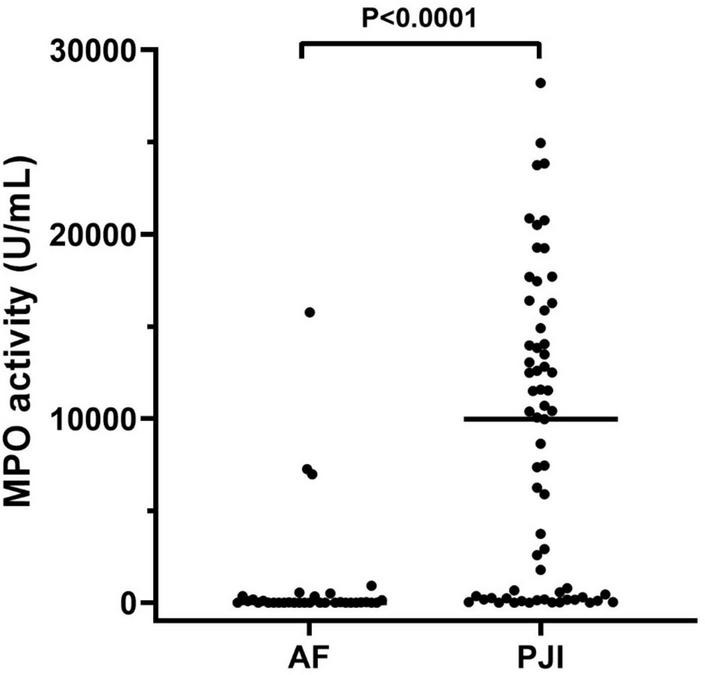
MPO activity measured in the synovial fluid of the enrolled population. The concentration of active MPO, reported as U/mL, was higher in subjects with periprosthetic joint infections (PJI, *n* = 65) than those with aseptic failure (AF, *n* = 34, *p* < 0.0001). In the graphs, the filled horizontal lines represent the median.

The results of active MPO measured in synovial fluids are showed in Figure 2. As displayed, active MPO was higher in PJI than AF (*P* < 0.0001, Figure 2) with a median value higher than 1400 times in PJI.

We evaluated whether MPO levels could be influenced by covariates such as sex, age, BMI and site of arthroplasty. Our findings indicate that the difference in MPO levels between PJI and AF remained consistent with the inclusion of all the covariates (*P* < 0.001). However, among the tested variable, only the site of arthroplasty was able to significantly influence the relationship between MPO and infection (its significance levels as a factor in ANCOVA analysis was *P* = 0.022, whereas for age was *P* = 0.9, sex *P* = 0.575, BMI P = 0.06). Further analysis, separating the site of arthroplasty in PJI and AF cases, revealed that MPO levels were significantly higher only in AF patients who underwent hip surgery rather than knee surgery (*P* = 0.022, see Supplementary Figure 1). To identify possible clues about this difference, we collected different pre-operative variables for a small subset of patients (*n* = 37, AF: *n* = 22, PJI: *n* = 15), including operative time, number of operators, white blood cells (WBC), neutrophil count, C-reactive protein (CRP). The results are summarized in Supplementary Table 1. Notably, knee and hip patients did not differ in operative time, thus ruling it out as a potential explanation for the higher MPO levels observed in hip surgery patients (120 min for hip vs. 95 min for knee, *P* = 0.216). Furthermore, all other variables showed no significant differences between knee and hip arthroplasty, except for neutrophils count, which was higher in knee arthroplasty compared to hip arthroplasty (*P* = 0.008).

**FIGURE 3:**
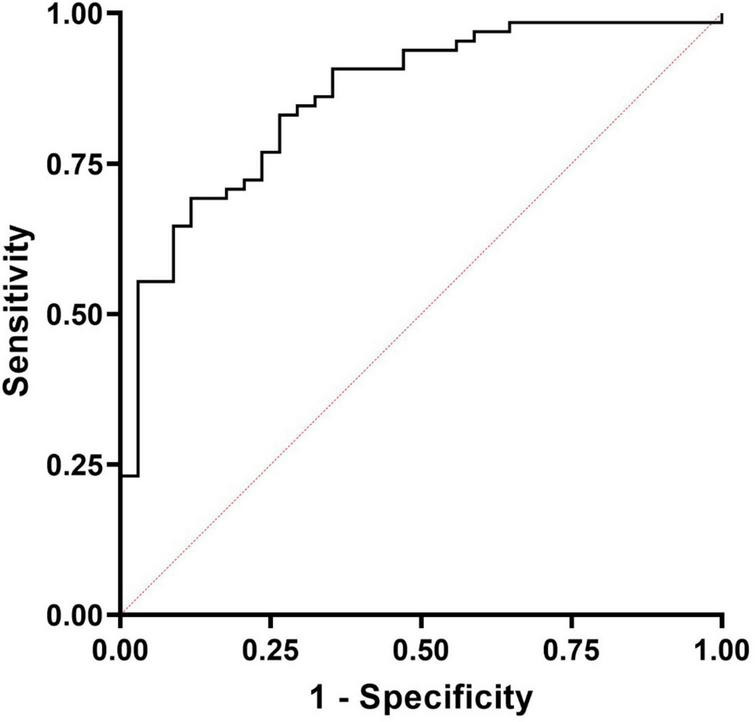
Receiver operating characteristic (ROC) curves for the diagnosis of PJI based on MPO levels measured in synovial fluids. The calculated AUC was significant (0.861; 95% CI: 0.786–0.935, *P* < 0.0001) and was able to correctly discriminate PJI from aseptic failures.

We then explored whether MPO was able to significantly discriminate between AF (control group) and PJI through a ROC analysis. The results are presented in Figure 3. MPO showed a significant Area Under the Curve (AUC, 0.861; 95% CI: 0.786–0.935, *P* < 0.0001) confirming its ability to distinguish between AF and PJI cases. By maximizing the Youden J index, we calculated a cut-off value of 561.9 U/mL, with good sensitivity (0.69) and specificity (0.88), and an accuracy of 75.76% (95% CI: 66.11–83.81%), a positive likelihood ratio of 5.88 (95% CI: 2.31–14.98) and a negative likelihood ratio of 0.35 (95%CI: 0.24–0.51), suggesting that subjects with a value of MPO higher than 561.9 had 5.88 times more probability of being infected.

**FIGURE 4:**
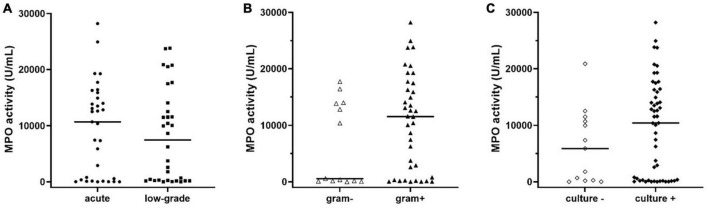
Levels of active MPO measured in the synovial fluid of subjects with PJI divided into acute or low-grade infection **(A)**, in gram-negative or gram-positive isolates **(B)**, or negative and positive cultural result **(C)**. The concentration of MPO was not different between acute or low-grade infections (panel **A**, *p* = 0.643). Subjects with gram-positive isolates showed a non-significant trend (panel **B**, *p* = 0.158) towards higher levels of active MPO when compared to those with gram-negative isolates. Subjects with a positive cultural result showed a trend towards higher levels of active MPO compared to negative cultural result (*p* = 0.183). In the graphs, the filled horizontal lines represent the median.

Finally, by inspecting the distribution of MPO in subjects with PJI (see Figure 2), we observed that a part of patients seemed to have extremely low levels of MPO compared to the median value (Figure 2, solid line within the graph). Thus, according to a previously published classification (Izakovicova et al., 2019) we separated PJI cases into acute (*n* = 33, 50.7%) and low-grade (*n* = 32, 49.3%) infections, to observe whether the levels of MPO could be influenced by this factor. As displayed in Figure 4A, the levels of active MPO were not different between the two groups (*P* = 0.643). We then analyzed whether the presence of gram-positive or gram-negative isolate was able to influence the levels of MPO, as previously stated in other papers (Gupta et al., 2022). As summarized in Figure 4B, subjects with a gram-positive bacterium showed a non-significant trend (*P* = 0.157) towards higher active MPO levels than those with gram-negative bacterium. Of note, negative or positive cultural tests did not show significantly different levels of active MPO (Figure 4C, *P* = 0.185), although subjects with a positive cultural isolate had a trend towards higher MPO.

### 3.3 Leukocyte esterase

The LE assay performed in our samples demonstrated a sensitivity of 50% (95%CI: 26–73) and a specificity of 85% (95%CI: 63–90), with an accuracy of 69% (95%CI: 52–82) and positive/negative LR of 3.50 (95%CI: 1.10–11) and 0.58 (95%CI: 0.36–0.96), respectively. Subjects being positive for LE had also significantly higher levels of MPO (*p* = 0.008). However, when subjects were separated based on diagnosis, those that were positive to LE did not show any significant difference from those with negative LE test (*p* = 0.307 within AF and *p* = 0.222 within PJI), probably due to sample size.

## 4 Discussion

The present study represents the first attempt to employ an activity assay to detect the active form of MPO in synovial fluid rather than measuring the protein mass. Even though reports of a fair correspondence between the two measures exist (Gach et al., 2015), it is possible that determining the active MPO or its specific activity, defined as the activity to mass ratio, will yield more illuminating findings. Furthermore, this is the first time this biomarker has been validated using the more widely accepted EBJIS criteria for the diagnosis of PJI.

Only two previous studies investigated the value of MPO as a diagnostic marker of PJI (Ikeda et al., 2020). In a recent paper authored by Ikeda S. et al., MPO was explored in synovial fluid from 37 patients of which only 19 suffered from chronic PJI, diagnosed with ICM criteria. MPO levels, detected with a conventional ELISA assay, were significantly higher in the synovial fluids of infected compared to non-infected patients with high sensitivity (100%) and specificity (94.4%) when using a cut-off value set at 16,463 ng/mL (Ikeda et al., 2020). In the second paper authored by Kimura et al., MPO was investigated through ELISA in 16 PJI cases diagnosed with ICM criteria, compared to 16 cases of AF. Patients with PJI had higher levels MPO (1436 ng/mL) than those in the non-PJI group with sensitivity of 94% and specificity of 100% (de Sandes Kimura et al., 2024). This finding, therefore, confirmed the results of Ikea et al., albeit with different cut-offs highlighting how diagnostic accuracy is highly variable depending on the type of ELISA kit employed. Furthermore, a paper published in 2022 showed how patients with PJI had a higher MPO content in microvesicles isolated from synovial fluid and detected by mass spectrometry investigation but with the limitation of not providing any potential diagnostic parameter (Sallai et al., 2022). The studies carried out on the diagnostic potential of PMO in PJI are summarized in Table 3.

**TABLE 3:** Investigation of MPO in the diagnosis of periprosthetic joint infection.

References	PJI Criteria	Patients with PIJ N.	MPO Detection Assay	MPO Diagnostic level
(Ikeda et al., 2020)	ICM	18	ELISA	82,125 ng/mL
(Sallai et al., 2022)	MSIS/ICM	17	Mass Spectrometry	n.a.
(de Sandes Kimura et al., 2024)	ICM	16	ELISA	1436 ng/μL

MSIS, Musculoskeletal Infection Society criteria; ICM, Second International Consensus Meeting on Musculoskeletal Infection criteria; n.a.: not available.

The proposed activity assay designed to detect the active isoform of MPO showed encouraging results in distinguishing PJI from AF (*P* < 0.0001) with an AUC greater than 0.8 and a sensitivity and specificity of 69% and 88%, respectively, when using a cut-off value of 561.9 U/mL. In addition, by setting this cut-off value, the test demonstrated a higher positive (5.88) and a low negative (0.35) likelihood ratio, confirming that subjects presenting with a value of active MPO, measured in the synovial fluid, greater than 561.9 u/mL have a 5.88 times higher probability to be positive for PJI. Of note, in the context of diagnostic tests performance, the use of likelihood ratios rather than positive/negative predictive values is more appropriate due to the impact of disease prevalence on the latter (Parikh et al., 2009).

It is noteworthy to mention that, in our hand, the LE assay demonstrated a rather low sensitivity of 50% and a specificity of 85%, with a modest accuracy (69%). This is likely attributable to limited size of the sub-population examined due to contamination of specimens with RBCs, as well as operator variability. In fact, it is already established that, despite its cost-effectiveness and speed of execution, the interpretation of rapid tests can be subjective, and the presence of different substances (e.g. RBCs) may render the test unfeasible (Colvin et al., 2015).

Collectively taken, our data support the idea that active MPO could be a reliable biomarker in detecting PJI, even better than LE.

In addition, we can assert the validity of the MPO for all patients, since we did not find that the confounding variables considered in our work (e.g. age, sex, BMI, site or arthroplasty) were able to significantly impact on the difference in MPO levels between PJI and AF. It is worth noting that patients undergoing hip arthroplasty exhibited higher MPO levels than those undergoing knee arthroplasty and were characterized by a decreased number of neutrophils. Therefore, we might speculate that in patients experiencing aseptic failure of a hip implant, there could be an increased recruitment of neutrophils at the hip joint. This may manifest as an increased production of MPO within the synovial fluid, accompanied by a slight decrease in the number of circulating neutrophils. However, this discrepancy appears to be masked by the underlying pathology when patients have a PJI.

However, we must exercise caution in interpreting these results due to the relatively small number of subjects. Even if statistically significant, the findings may lack of generalizability and thus applicability to the broader population.

A possible explanation for the decrease in the diagnostic performance of the proposed activity assay compared to earlier results obtained with the conventional ELISA could lie in several reasons: first and foremost, the fact that the test quantifies MPO enzyme activity rather than protein mass. Therefore, the two essays are not directly comparable; second, the sample size. In fact, in our study 65 PJI samples were analyzed compared to the 19 cases previously described; third, the use of the EBJIS diagnostic algorithm, which traditionally has shown less diagnostic consistency in the evaluation of other biomarkers (Vale et al., 2023).

Interestingly, when comparing MPO levels between “acute” (*n* = 33) and “low-grade” (*n* = 32) PJI, no statistically significant difference was found (Figure 4A). This result would give active MPO a non-negligible diagnostic potential even in PJI sustained by low levels of inflammation, a category in which the biomarkers tested so far are known to lose some of their effectiveness.

Comparison of the gram-positive and gram-negative subgroups of PJIs revealed no statistically significant difference in active MPO levels. Nevertheless, a trend towards higher MPO levels was observed in cases where the etiologic agent was a gram-positive bacterium (Figure 4B). The lack of statistical significance of our result could be attributable to the smaller frequency of gram-negative infections compared to gram-positive ones.

Nonetheless, the increased trend in MPO release within the synovial fluid observed in the presence of gram-positive bacteria aligns with previous studies, indicating that various bacterial species can influence NET formation and composition (Dwyer et al., 2014; Gupta et al., 2022). However, it is still a matter of debate to what extent various bacteria can modulate NET composition, and what impact this modulation could have on NET function. Thus, it is tempting to speculate that gram-positive bacteria may drive the NET formation towards a more MPO-richer environment in the joint. However, this aspect has never been specifically investigated or confirmed in the joint environment during infection, in vivo or in vitro, where the synovial fluid components could even impact biofilm formation and help the microorganism to evade NET defense (Pestrak et al., 2020).

Several biomarkers derived from neutrophils, including leukocyte esterase (LE), alpha-defensin and calprotectin, have recently been evaluated as an aid in the diagnosis of PJI (Renz et al., 2018; Grassi et al., 2022; Schindler et al., 2023). However, their diagnostic performance, is highly variable depending on the chosen PJI definition, with a poorer consistency with EBJIS criteria compared to Musculoskeletal Infection Society (MSIS) algorithm (Vale et al., 2023). Moreover, the lack of standardized laboratory methods and universally accepted threshold values make their use difficult in clinical practice (Hantouly et al., 2022).

This statement is significant since the various PJI definition criteria are not unanimously recognized but they could significantly impact the assessment of novel diagnostic test performance.

The urine LE strip test has been suggested in several papers for the purpose of screening synovial fluid in patients with PJI. Although widely accessible and reasonably priced, this biomarker has been shown to have low sensitivity in different studies, primarily due to blood contamination of the sample and the highly subjective interpretation of the test’s color reading (Colvin et al., 2015).

The highest reported accuracy for PJI diagnosis was found with the alpha defensin ELISA test. In 2019, the FDA authorized the employment of a lateral flow quick test version of the alpha-defensin assay (SynovasureTM) for the diagnosis of PJI (Deirmengian et al., 2021). Although this qualitative test may be useful in the intraoperative setting due to its ability to provide results within 10 min without specialized equipment, its cost is high and the test’s sensitivity proved to be limited (54% to 84%). Therefore, its use as a screening tool for PJI is not recommended (Renz et al., 2018).

Calprotectin has recently been successfully studied as a diagnostic biomarker in PJI, and attracted interest because of its low cost (Grassi et al., 2022).

Multiple laboratory methods, such as immunoturbidimetric immunoassays, lateral flow tests, and ELISAs, have been investigated to identify calprotectin in synovial fluid, with promising results (Salari et al., 2020; Grzelecki et al., 2021). However, its use in routine diagnostics is limited by several reasons including the heterogeneity of laboratory technique, the lack of standardized quantitative cut-offs, small number of patients enrolled in each study, the discrepancy of the results in relation to the site of the affected joint (hip, knee and shoulder prosthesis), and dissimilar performance of the test with the different criteria used for the diagnosis of PJI (Hantouly et al., 2022).

Neutrophils are crucial players in the pathogenesis of PJI. Unlike previously thought, neutrophils are not terminal cells that cannot change after leaving the bone marrow. For instance, bacterial products can significantly increase their antimicrobial activities (Kobayashi et al., 2023).

Recent research has shown that their involvement in the pathogenesis of PJI could take place through the formation of neutrophil extracellular traps (NETosis) (Cai et al., 2023). NETosis reflects part of the innate immune response to pathogens. By localizing proteases and breaking down cytokines and chemokines, NETs can trap and eliminate microorganisms while preventing collateral harm (Yipp et al., 2012; Schauer et al., 2014).

NETosis is the process by which neutrophils produce and release decondensed chromatin fibers encapsulating granular enzymes, also known as NETs. Bacteria and fungi are among the microbes that can be ensnared by NETosis, which was initially reported in 2004 (Brinkmann and Zychlinsky, 2012). NETs-complexes of histone proteins and DNA coated with proteolytic enzymes are produced extracellularly to trap infections and facilitate their removal (Meier et al., 2024).

According to previous studies, severe MPO deficit is primarily associated with recurring fungal infections (Papayannopoulos, 2018) and critical function of NETs has been proven in combating pathogens, including fungal hyphae, that are too big to be eliminated intracellularly (He et al., 2022).

These findings would make NETosis potentially valuable in the diagnosis of peri-prosthetic fungal infections, although the presence of only one patient with fungal PJI in our case series makes this statement a mere speculation.

Neutrophil elastase, MPO, calprotectin, cathelicidins and defensins were the first NETs components to be identified. This list has been expanded by further research, which indicates that different stimuli are able to affect the structure of NETs (Papayannopoulos, 2018), although future studies will be needed to expand Knowledge on NET’s function in the context of PJI.

This pilot study has advantages and limitations. Undisputed advantages lie in an accurate and reproducible result. The procedure is readily adaptable and easily repeatable in any laboratory. This test could be seen as time and money-saving, since less than 10 Euros was spent for a single test.

Limitations include discrepancy in the numbers of PJI vs. AF, validation of the test with a single diagnostic algorithm and the time required for the performance of the technique.

## 5 Conclusion

The proposed assay appears to be a reliable and affordable tool ( < 10 Euros/test) for detecting the active MPO in synovial fluid, with promising characteristics of sensitivity and specificity in discriminating both acute and low-grade PJI from AF. Further studies are needed to confirm MPO diagnostic cut-off values and validate their use in the routine clinical practice in independent cohorts.

## Data availability statement

The raw data supporting the conclusions of this article will be made available by this authors, without undue reservation.

## Ethics statement

The studies involving humans were approved by the Institutional review boards of Charité Center for Musculoskeletal Surgery, Berlin; Casa di Cura Santa Maria Maddalena, Occhiobello, Italy. The studies were conducted in accordance with the local legislation and institutional requirements. The participants provided their written informed consent to participate in this study.

## Author contributions

MM: Writing−original draft, Writing−review and editing, Conceptualization, Methodology, Project administration. GD: Writing−review and editing, Methodology. VR: Writing−review and editing, Methodology, Validation. CC: Writing−review and editing. MCM: Writing−review and editing, Formal analysis. GZ: Writing−review and editing. RD: Writing−review and editing. MG: Writing−review and editing, Formal analysis. AC: Writing−review and editing, Methodology. CC: Writing−review and editing. YN: Writing−review and editing, Methodology, Formal analysis. AnT: Writing−review and editing. AlT: Writing−review and editing, Methodology, Project administration.
