# Supplementary material for: Active myeloperoxidase: a promising biomarker to differentiate “acute” and “low-grade” peri-prosthetic joint infections from aseptic failures
Source: Front Microbiol. 2024 Jun 7;15:1417049. doi: 10.3389/fmicb.2024.1417049 (PMC11190362; doi:10.3389/fmicb.2024.1417049)
Supplement: Supplementary file 1 [file Data_Sheet_1.pdf]

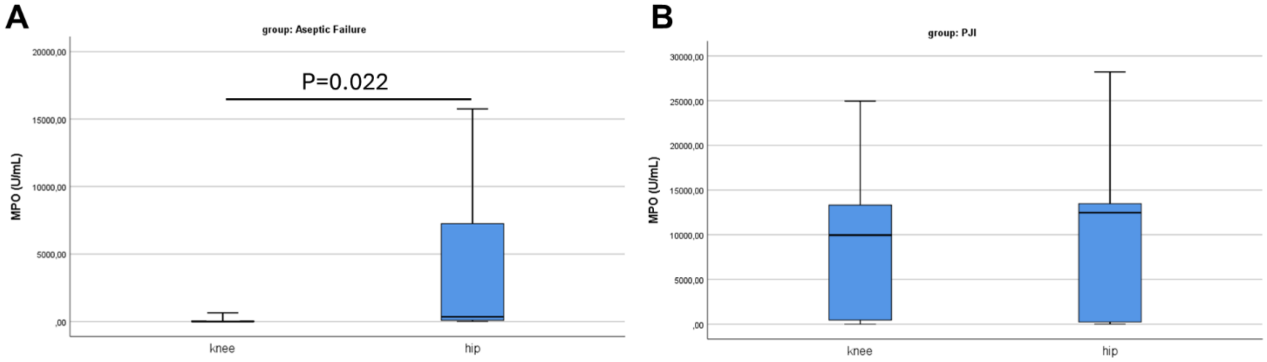

Supplementary Figure 1. MPO levels in Aseptic Failure and PJI patients separated by site of arthroplasty. Patients with hip arthroplasty experienced higher levels of synovial fluid MPO than those undergoing knee arthroplasty (P=0.008).

Supplementary Table 1. Pre-operative clinical variables of a subgroup of patients (n=37) included in the study, divided by site of arthroplasty.

	**Aseptic Failure (n=22)**		**PJI (n=15)**	
	Hip Arthroplasty (n=6; median (IQR))	Knee Arthroplasty (n=16	Hip Arthroplasty (n=4)	Knee Arthroplasty (n=11)
**Operative time (minutes)**	120 (83-160)	90 (80-120)	62 (51-103)	75 (73-80)
**Number of operators**	3 (2-4)	3 (2-4)	3 (3-4)	3 (2-3)
**White blood cells (cells/ µl)**	4495 (3530-6795)	6240 (5700-7840)[a]	7170 (6275-9825)	7490 (6520-9470)
**Neutrophils (cells/µl)**	2640 (2460-3407)	3850 (3610-5605)[b]	4720 (4100-7030)	4615 (4070-4955)
**CRP (mg/L)**	6.45 (1.12-10.7)	2.3 (0.67-6.0)	1.4 (0.95-30.3)	2.5 (0.95-14.2)
**Synovial fluid MPO (U/mL)**	349 (67-9375)	9.2 (0.5-112.2)[b]	12822 (3477-15561)	11570 (164-14907)

[a] P<0.05 vs. Hip arthroplasty
[b] P<0.01 vs. Hip arthroplasty
CRP, C-reactive protein
